# A nested case–control study of the association of *Helicobacter pylori* infection with gastric adenocarcinoma in Korea

**DOI:** 10.1038/sj.bjc.6602467

**Published:** 2005-03-08

**Authors:** A Shin, H R Shin, D Kang, S K Park, C-S Kim, K-Y Yoo

**Affiliations:** 1 Department of Preventive Medicine, Seoul National University College of Medicine, 28 Yongon-dong, Chongno-gu, Seoul 110-799, Republic of Korea; 2 Division of Cancer Control and Epidemiology, National Cancer Center Research Institute, 809 Madu1-dong, Ilsan-gu, Goyang-si, Gyeonggi-do 411-769, Republic of Korea; 3 Department of Preventive Medicine, Konkuk University College of Medicine, 322 Danwol-Dong, Chungju, Chungcheongbuk-Do 380-701, Republic of Korea

**Keywords:** gastric cancer, *Helicobacter pylori*, risk factors, nested case–control study, Korean

## Abstract

In a nested case–control study of 86 cases of gastric adenocarcinoma in relation to *Helicobactor pylori* infection in the Korean Multi-center Cancer Cohort, the *H. pylori* IgG seropositivity was 83.7% and that of the 344 matched controls was 80.8%, with a matched odds ratio for *H. pylori* infection of 1.06 (95% CI, 0.80–1.40).

In 1994, the International Agency for Research on Cancer (IARC) classified *Helicobactor pylori* as a group I carcinogen (IARC, 1994), whereas that same year, a US body concluded that there was insufficient evidence for a causal association between *H. pylori* infection and gastric adenocarcinoma (NIH Consensus Development Panel, 1994). During the last decade, several nested case–control studies of *H. pylori* infection and gastric adenocarcinoma, including a meta-analysis (Huang *et al*, 1998; Danesh, 1999; Eslick *et al*, 1999; Helicobacter and Cancer Collaborative Group, 2001), were carried out. In high *H. pylori* infection areas, such as Eastern Asia and Africa, the findings from epidemiological studies have shown inconsistent results (Miwa *et al*, 2002; Tajima, 2002; Lunet and Barros, 2003).

Gastric cancer is the cancer with the most frequent incidence and is the second most common cause of cancer death in Korea (Korea Central Cancer Registry, 2002; National Statistical Office, 2002), where the prevalence of *H. pylori* infection is also high. In view of its potential importance, we carried out a nested case–control study of the subjects in a Korean cancer cohort.

## MATERIALS AND METHODS

The Korean Multi-center Cancer Cohort (KMCC) consisted of male and female subjects aged over 30 years, who were voluntary participants in a cancer-screening survey in four geographically defined areas of Korea (Yoo *et al*, 2002). Each participant completed a detailed questionnaire administered by trained interviewers. In total, 10 ml of blood collected was fractionated and all the samples were stored at a temperature of −70°C. Informed consent was obtained from all the participants and the study protocol was approved by the Institutional Review Board of the Seoul National University Hospital and National Cancer Center.

Cases of cancer were identified through record linkage with the Korean Central Cancer Registry and the National Health Insurance Cooperation database. To validate the cancer diagnosis and obtain additional detailed clinical information, a medical record review was undertaken for potential gastric cancer patients. Among the 10 927 participants recruited between 1993 and 1999, 228 participants who had cancer prior to recruitment were excluded from the study. Among the remaining 10 699 potential study population, 86 incident gastric cancer cases were identified. The average time interval between the blood collection and the diagnosis of gastric cancer was 2.6 years. Four controls from the eligible cancer-free cohorts were matched to each cancer case by incidence density sampling based on their age within 5 years, gender, and the year and site of their recruitment. Thus, 86 newly diagnosed gastric adenocarcinoma patients and 344 matched controls were included in the final analysis.

A Genedia™ (Greencross Life Science) *H. pylori* IgG enzyme-linked immunosorbent assay (ELISA) kit, with a sensitivity of 100% and a specificity of 81.3% in the Korean population (Eom *et al*, 2001), was used to determine seropositivity of the cases and controls according to the manufacturer's protocol.

A multivariate conditional logistic regression model was used to calculate the adjusted matched odds ratio (OR) and 95% confidence intervals. Statistical analysis was performed with the SAS v8.1 statistical package.

## RESULTS

The demographic and clinical characteristics of the gastric adenocarcinoma cases are shown in Table 1; their mean age was 63 years at recruitment and 66% of the cases were male. Most of cases developed adenocarcinoma in the non-cardia region of the stomach (six cardia, 70 non-cardia, one both cardia and non-cardia, nine unspecified).

Cumulative smoking of more than 26 pack-years (OR, 1.22; 95% CI, 0.88–1.67) and a history of gastritis or gastric ulcer were not associated with increased risk (OR, 1.15; 95% CI, 0.80–1.67). Frequent consumption of yellow–green vegetables showed a decreased risk of gastric cancer, which was not significant (OR, 0.66; 95% CI, 0.35–1.27).

Overall, 72 (83.7%) of the 86 gastric cancer patients and 278 (80.8%) of the 344 controls were *H. pylori* seropositive (Table 2). Adjustment for other variables caused little change in the crude OR, but adjusted ORs only are reported. The matched OR for *H. pylori* infection to gastric cancer was 1.06 (95% CI, 0.80–1.40), slightly greater among younger age groups (40 and 50 years), but not significantly so (OR, 1.25; 95% CI, 0.73–2.14). There were no differences in risk between male and female subjects or by area of recruitment. A nonsignificant increased risk of gastric cancer associated with *H. pylori* positivity was observed among subjects with more than 5 years of follow-up (OR, 1.26; 95% CI, 0.64–2.48). When cases were stratified by cancer subsites, the risk decreased nonsignificantly (OR, 0.88; 95% CI, 0.38–2.28).

## DISCUSSION

The present study suggests that there might be no direct association between *H. pylori* infection and gastric adenocarcinoma risk in South Korea.

The confidence intervals of our study (0.80–1.40) are exclusive of those in the meta-analysis from 12 nested case–control studies (Helicobacter and Cancer Collaborative Group, 2001). The major heterogeneity among the studies in the meta-analysis was from age and time interval between sample collection and cancer diagnosis. There was a 2.4-fold (95% CI, 1.82–3.12) increase in risk when samples were collected less than 10 years before the diagnosis of cancer, as were our own. A Taiwanese study, which had a median 2.0 years of follow-up showed 1.55-fold nonsignificantly increased risk (95% CI, 0.68–3.54), whereas a Japanese study and a Chinese study with median 3.6 years of follow-up showed 3.38 (95% CI, 1.15–9.90)- and 1.66 (95% CI, 1.08–2.54)-fold increased risk, respectively (Webb *et al*, 1996; Watanabe *et al*, 1997; Yuan *et al*, 1999). A recent Japanese study with 4.2 years of follow-up suggested an increased risk in women with *H. pylori* infection (OR, 2.6; *P*=0.013), but not in men (Yatsuya *et al*, 2004). Therefore, our result might be a consequence of a shorter period between blood sampling and cancer diagnosis. However, we could not find significant differences of ORs in different time intervals in our data. The magnitude of the association was much greater among subjects aged less than 60 years than among those aged 60 years or more in the meta-analysis. In our data, 69% of the patients were aged 60 years or older; therefore, the magnitude of the association also might be smaller than in other studies.

Two nested case–control studies conducted in Asian countries showed less than a two-fold increased risk, and one study showed a 3.4-fold increased risk (Webb *et al*, 1996; Watanabe *et al*, 1997; Yuan *et al*, 1999). In addition, a Finnish study in which the seropositivity of the control group was greater than 80%, also showed only a 1.5-fold nonsignificantly increased risk (Aromaa *et al*, 1996). Compared with western countries, most Asian and African countries showed higher infection rates of *H. pylori*, but their gastric cancer incidence rates vary markedly, perhaps partially explained by inaccurate data and the interactive effects of fruits and alcohol consumption (Lunet and Barros, 2003).

The major limitations of our study are its relatively small size of the cohort and short follow-up period, and a larger study with longer follow-up period will be needed to confirm its findings in this study region. The study participants were mostly farmers, so a generalisation with respect to the entire Korean population cannot be assumed. The disappearance of incidence of childhood infection may have introduced temporality bias (Ohata *et al*, 2004). The history of gastric ulcer or gastritis did not affect the association of *H. pylori* infection to gastric cancer in our data (data not shown). The use of serum pepsinogen as a marker of chronic gastritis could usefully be applied in a future study.

## Figures and Tables

**Table 1:** Demographic and clinical characteristics of gastric adenocarcinoma cases in the nested case–control study

**Characteristics**	**Categories**	**Number**	**(%)**
Age (years)	40–59	27	(31.4)
	60–69	41	(47.7)
	70–82	18	(20.9)

Sex	Male	57	(66.3)
	Female	29	(33.7)

Areas at recruitment	Haman county	50	(58.2)
	Choongju city	26	(30.2)
	Uljin county	5	(5.8)
	Youngil county	5	(5.8)
			
Tumour sites	Cardia	6	(7.0)
	Non-cardia	70	(81.3)
	Both	1	(1.2)
	Unspecified	9	(10.5)

Follow-up duration	<1 year	24	(27.9)
	1–2 years	33	(38.4)
	3–5 years	16	(18.6)
	>5 years	13	(15.1)

Total		86	(100.0)

**Table 2:** Seropositivity for *Helicobacter pylori* IgG antibody and adjusted matched odds ratios (ORs) for their relationship stratified by clinical characteristics and demographics

**Strata**	**Cases**		**Controls**		**Adjusted matched OR^b^ (95% CI)**
	**Infected/total**	**Hp^a^ (+), %**	**Infected/total**	**Hp^a^ (+), %**	
Overall	72/86	(83.7)	278/344	(80.8)	1.06 (0.80–1.40)

*Tumour sites*					
Cardia	4/6	(66.7)	20/24	(83.3)	0.88 (0.38–2.28)
Non-cardia	60/70	(85.7)	231/280	(82.5)	1.07 (0.77–1.49)

*Follow-up duration*					
<1 year	21/24	(87.5)	80/96	(83.3)	1.10 (0.63–1.92)
1–3 year	27/33	(81.8)	109/132	(82.6)	1.02 (0.63–1.65)
3–5 years	12/16	(75.0)	51/64	(79.7)	0.96 (0.51–1.81)
>5 years	12/13	(92.3)	38/52	(73.1)	1.26 (0.64–2.48)

*Age (years)*					
40–59	25/27	(92.6)	88/108	(81.5)	1.25 (0.73–2.14)
60–69	33/41	(80.5)	135/164	(82.3)	0.97 (0.64–1.47)
70–82	14/18	(77.8)	55/72	(76.4)	1.03 (0.59–1.77)

*Sex*					
Male	47/57	(82.5)	179/228	(78.5)	1.08 (0.78–1.50)
Female	25/29	(86.2)	99/116	(85.3)	1.03 (0.60–1.77)

*Recruited areas*					
Haman county	41/50	(82.0)	159/200	(79.5)	1.05 (0.74–1.49)
Choongju city	22/26	(84.6)	85/104	(81.7)	1.06 (0.62–1.81)
Uljin county	5/5	(100.0)	18/20	(90.0)	1.03 (0.18–6.02)
Youngil county	4/5	(80.0)	16/20	(80.0)	1.06 (0.29–3.84)

a Hp (+)=*Helicobacter pylori* seropositivity.

b Adjusted matched OR controlling for education, alcohol consumption, and cumulative dose of smoking.
